# Next-generation molecular tools in veterinary parasitology: advances, challenges, and perspectives in the diagnosis of emerging parasites

**DOI:** 10.1590/S1984-29612026023

**Published:** 2026-07-06

**Authors:** Tayná Padilha Basqueroto Antunes, Edward Antunes

**Affiliations:** 1 Michigan State University, College of Veterinary Medicine, Veterinary Diagnostic Laboratory, Lansing, MI, United States of America; 2 Faculdade Iguaçu, Programa de Pós-graduação em Finanças e Gestão Hospitalar, Capanema, PR, Brasil

**Keywords:** Molecular diagnostics, next-generation sequencing, metagenomics, emerging parasites, epidemiological surveillance, zoonoses, Diagnóstico molecular, sequenciamento de nova geração, metagenômica, parasitas emergentes, vigilância epidemiológica, zoonoses

## Abstract

Advances in molecular technologies have revolutionized veterinary parasitology, providing highly sensitive and specific tools for the detection, characterization, and surveillance of parasites in domestic and wildlife species. Approaches such as next-generation sequencing, metabarcoding, and metagenomics have significantly enhanced the ability to identify previously unknown or uncultivable species, detect complex coinfections, and deepen our understanding of parasite genetic diversity, evolution, and population dynamics. Beyond their impact on laboratory diagnostics, these tools have proven essential for the early detection of zoonoses, environmental monitoring, and the development of integrated surveillance systems under the One Health framework. This review synthesizes the major technological advances and their practical applications in both global and Latin American contexts, particularly Brazilian, highlighting how the incorporation of these tools has the potential to transform strategies for surveillance, prevention, and response to emerging and re-emerging parasitic diseases. Challenges related to standardization, cost, infrastructure, and technology transfer are also discussed, along with future perspectives for large-scale implementation aimed at strengthening diagnostic capacity and epidemiological surveillance in the face of increasing parasitic threats in a rapidly changing world.

## Resumo

Os avanços nas tecnologias moleculares têm revolucionado a parasitologia veterinária, proporcionando ferramentas altamente sensíveis e específicas para a detecção, caracterização e vigilância de parasitos em espécies domésticas e silvestres. Abordagens como o sequenciamento de nova geração, o *metabarcoding* e a metagenômica ampliaram significativamente a capacidade de identificar espécies anteriormente desconhecidas ou não cultiváveis, detectar coinfecções complexas e aprofundar a compreensão sobre a diversidade genética, evolução e dinâmica populacional dos parasitos. Além do impacto no diagnóstico laboratorial, essas ferramentas têm se mostrado essenciais para a detecção precoce de zoonoses, o monitoramento ambiental e o desenvolvimento de sistemas de vigilância integrados no contexto do conceito *One Health*. Esta revisão sintetiza os principais avanços tecnológicos e suas aplicações práticas, tanto no cenário global quanto no contexto latino-americano, em especial o brasileiro, destacando como a incorporação dessas ferramentas tem potencial para transformar estratégias de vigilância, prevenção e resposta frente a doenças parasitárias emergentes e reemergentes. Também são discutidos os desafios relacionados à padronização, custos, infraestrutura e transferência de tecnologia, além das perspectivas futuras para sua implementação em larga escala, com o objetivo de fortalecer a capacidade diagnóstica e a vigilância epidemiológica diante das crescentes ameaças parasitárias em um mundo em rápida transformação.

## Introduction

Parasitic diseases remain a significant challenge for veterinary medicine and public health, with impacts that extend far beyond animal health. These infections compromise animal welfare, reduce livestock productivity, and directly affect the economy of rural communities, threatening the livelihoods of populations that depend on animal husbandry and wildlife (Matoute et al., 2024). Although their true extent is difficult to estimate, parasites are known to be widely distributed and continue to contribute substantially to global sanitary and economic burdens (Lymbery & Thompson, 2012; Pisarski, 2019).

In recent decades, factors such as climate change, increased international trade, and changes in land use have profoundly influenced the distribution and diversity of parasites. As a result, these organisms are expanding into new geographic areas and infecting hosts where they were not previously reported (Caminade et al., 2019; Torgerson & Macpherson, 2011).

Molecular technologies have emerged as powerful solutions to these challenges, transforming the way parasitic infections are detected, characterized, and monitored. Over the past two decades, next-generation sequencing (NGS), metabarcoding, and metagenomics have been successfully applied in various parts of the world, including North America, Europe, and Asia (Miller et al., 2024). These approaches have enabled the detection of previously unrecognized or uncultivable species, revealed complex coinfections, and provided novel insights into parasite diversity, population structure, and evolutionary dynamics (Avramenko et al., 2015). Additionally, digital polymerase chain reaction (dPCR) has proven valuable for the precise quantification of parasitic DNA and the monitoring of antiparasitic resistance, contributing to more effective disease control strategies (Elmahalawy et al., 2018).

While molecular technologies have revolutionized parasitology worldwide by enhancing parasite detection, improving surveillance, and generating new insights into parasite diversity, epidemiology, and resistance, their development, implementation, and impact remain uneven across regions. This disparity is particularly evident in low- and middle-income regions such as Latin America, where structural and financial barriers, including limited funding, inadequate infrastructure, the need for specialized technical training, and the absence of standardized protocols, continue to hinder large scale implementation in countries such as Brazil (Cunha et al., 2019; Ferreira et al., 2020).

Despite these challenges, recent studies demonstrate the transformative potential of molecular approaches in the region. Nemabiome/ITS2 metabarcoding has been applied in cattle, including samples from São Paulo State, Brazil, revealing the composition of nematode communities and generating data useful for diagnosis and surveillance (Avramenko et al., 2017). Molecular characterization of protozoa in ruminants has also been reported in Brazil, highlighting the utility of genetic tools for detection and genotyping (Holsback et al., 2023).

In the South American Amazon, next generation sequencing based metabarcoding approaches have also demonstrated their capacity to uncover parasites of veterinary importance in wildlife, indicating the potential for methodological transfer to livestock systems(Matoute et al., 2024).

This review aims not only to summarize recent advances in molecular technologies applied to veterinary parasitology, but also to critically position these tools within the specific context of Latin America, particularly Brazil. While several reviews have addressed the technical performance and applications of molecular approaches, fewer studies have explored the practical challenges associated with their implementation in low- and middle-income settings.

Therefore, this review highlights the regional disparities in access to molecular diagnostics, infrastructure limitations, and barriers related to cost, training, and standardization. By integrating global advances with region-specific realities, this work provides a contextualized perspective on how molecular tools can be effectively translated into practice in Latin America, contributing to more equitable and sustainable One Health surveillance systems.

## Material and Methods

### Studying design

This study was designed as a scoping review aiming to systematically map, summarize, and synthesize the available scientific evidence on the application of molecular tools in veterinary parasitology. This approach is appropriate for exploring broad research areas, identifying key concepts, methodological trends, and knowledge gaps in the literature. The review focuses on global advances as well as the specific context of Latin America, particularly Brazil, highlighting practical applications, limitations, and future perspectives associated with these technologies.

### Descriptors and databases

The literature search was conducted between January and September 2025 using four major scientific databases: PubMed, Scopus, Web of Science, and SciELO. These databases were selected due to their broad coverage of biomedical, veterinary, and parasitology literature, ensuring the inclusion of studies from both global and Latin American contexts.

The search employed controlled descriptors and free terms related to molecular diagnostics and veterinary parasitology in English, Portuguese, and Spanish. The main descriptors included molecular diagnosis, molecular tools, veterinary parasitology, animal parasites, parasitic infections, metabarcoding, metagenomics, NGS, digital PCR, Latin America, Brazil, wildlife, and livestock.

Reference lists of relevant articles were also manually screened to identify additional studies not retrieved through the database searches.

### Inclusion and exclusion criteria

The inclusion criteria were:

- Original articles, reviews, and book chapters published between 2000 and 2025**.**
- Studies addressing the use of molecular tools in the detection, identification, characterization, or surveillance of parasites in veterinary contexts.
- Research conducted worldwide, with special attention to studies carried out in Latin America and Brazil**.**
- Articles written in English, Portuguese, or Spanish**.**

Exclusion criteria included:

- Studies focusing exclusively on human parasitology without veterinary relevance.
- Publications lacking sufficient methodological detail or not directly related to molecular approaches.
- Non-peer-reviewed sources such as conference abstracts, opinion pieces, or unpublished theses.

### Search strategy

A descriptive analysis was conducted to highlight both global developments and regional applications, with special emphasis on studies carried out in Latin America, particularly Brazil. Boolean operators AND and OR were used to combine the descriptors and refine the search results. An example of the search string applied in the databases was:

("molecular diagnosis" OR "molecular tools" OR "molecular techniques") AND ("veterinary parasitology" OR "animal parasites" OR "parasitic infections") AND ("next generation sequencing" OR "NS" OR "metabarcoding" OR "metagenomics" OR "digital PCR").

All retrieved references were imported into reference management software to organize the records and facilitate duplicate removal.

### Data extraction and analysis

For each included study, key information was extracted using a structured approach. The variables collected included: authors, year of publication, geographic location, host species, target parasite(s), molecular methods used, main findings, and relevance to veterinary parasitology. The data were organized thematically to identify methodological trends, technological advances, and knowledge gaps.

### Study selection process

The study selection process followed the PRISMA-ScR (Preferred Reporting Items for Systematic Reviews and Scoping Reviews) (Tricco et al., 2018) to ensure transparency and reproducibility. After duplicate removal, titles and abstracts were screened according to predefined inclusion and exclusion criteria to assess their relevance. Articles considered potentially eligible were subsequently evaluated through full text assessment.

A total of 312 records were initially identified through database searches and additional sources, including 208 studies published in English, 74 in Portuguese, and 30 in Spanish, representing the total number of records retrieved across all three languages. After removing 60 duplicate records, 252 records remained for title and abstract screening. Of these, 146 studies were selected for full-text evaluation. Following the inclusion and exclusion criteria described above, 78 studies were deemed eligible for analysis and included in the final synthesis. The complete study selection workflow is illustrated inFigure 1. As this study was designed as a scoping review, a formal risk-of-bias assessment was not performed, as the primary objective was to map the existing literature rather than to evaluate the methodological quality of individual studies.

**Figure 1:**
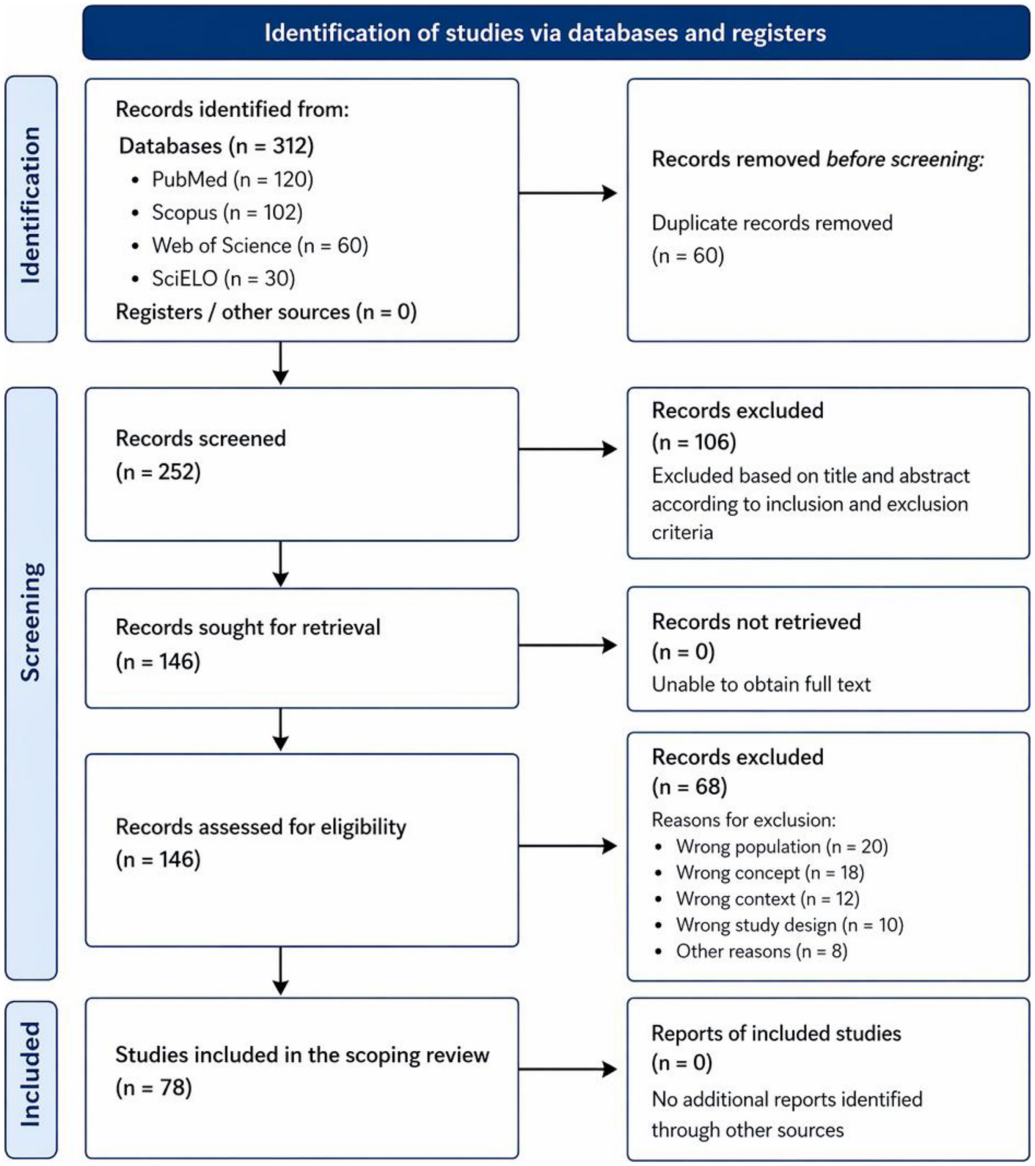
Overview of Literature Identification and Eligibility Assessment. Source: personal archive.

## Results and Discussion

### Molecular epidemiology and genetic diversity using next-generation sequencing

Molecular epidemiology has emerged as one of the most powerful strategies for understanding the evolutionary dynamics, transmission, and spread of parasitic diseases in veterinary medicine (Martínez et al., 2024; Ye et al., 2025).Unlike traditional epidemiological methods, which rely on clinical patterns, ecological observations, or phenotypic identification, molecular epidemiology integrates genetic information to uncover hidden transmission networks, cryptic species, and population structure (Lymbery & Thompson, 2012). This integration is especially relevant for parasites with complex life cycles, multiple intermediate hosts, or zoonotic potential, which often evade detection by classical tools (Santa et al., 2021).

Molecular markers constitute the foundation of modern epidemiological inference. Mitochondrial genes such as*cox1*and*nad1*are frequently used in phylogeographic studies because of their relatively high mutation rate and maternal inheritance, enabling the discrimination between parasite strains and geographic lineages (Kern et al., 2020; Papaiakovou et al., 2023). Ribosomal markers such as ITS1, ITS2, and 18S rRNA are widely applied in species identification, metabarcoding, and genotyping due to their conserved flanking regions and multicopy nature (Chan et al., 2021). Markers with higher discriminatory power, such as microsatellites and single nucleotide polymorphisms (SNPs), allow fine-scale analyses of population structure and gene flow, which are essential for outbreak investigations and the detection of resistance-associated variants (Chaudhry et al., 2016; Frantz et al., 2023; Nair et al., 2014; Kim et al., 2022). The principal molecular markers used in veterinary parasitology, together with their applications, advantages, and limitations, are presented in Table 1.

**Table 1:** Molecular markers and their applications in veterinary molecular epidemiology.

**Molecular marker / technique**	**Common targets**	**Applications**		**Advantages**	**Limitations**	**References**
Mitochondrial genes (*cox1*, *nad1*)	Helminths, protozoa	Phylogeography, strain differentiation		High mutation rate, robust amplification	Maternal inheritance only	(Kern et al., 2020; Papaiakovou et al., 2023)
Ribosomal DNA (ITS1, ITS2, 18S)	Protozoa, helminths	Species ID, metabarcoding, genotyping	Highly conserved primers, multicopy		Lower resolution in closely related taxa	(Chan et al., 2021)
Microsatellites	*Leishmania*, *Toxoplasma*, nematodes	Population structure, outbreak tracing		Higher discriminatory power	Requires prior marker development	(Chaudhry et al., 2016; Frantz et al., 2023)
SNP genotyping / MLST	Multiple parasites	Resistance detection, transmission studies		High resolution, portable datasets	Bioinformatics dependent	(Nair et al., 2014; Kim et al., 2022)
Metabarcoding (NGS)	Mixed infections (e.g., *Eimeria*)	High throughput diagnostics, co-infection detection		Multiplex capacity, rapid	Reference bias, data processing complexity	(Papaiakovou et al., 2023; Martínez et al., 2024)
WGS	Protozoa, helminths	Population genomics, phylogenomics, resistance monitoring		Full genome resolution, discovery of novel loci	Higher cost, infrastructure requirements	(Martínez et al., 2024; Ye et al., 2025)

Molecular epidemiological analyses have also clarified transmission patterns in several parasites of veterinary and zoonotic importance. Studies of*Trypanosoma cruzi*using minicircle DNA markers, SNPs, and whole-genome approaches have identified distinct domestic and sylvatic transmission clusters and multiple transmission events (Llewellyn et al., 2009). Similarly, analyses of*Leishmania infantum*based on SNPs, kDNA markers, and genomic data have revealed regional population structuring, clonal expansion, and the emergence of drug-resistant variants (Carnielli et al., 2018).

Multilocus genotyping of*Toxoplasma gondii*has also identified major clonal lineages and recombination hotspots in different geographic regions (Lorenzi et al., 2016). Additional representative examples of molecular epidemiology studies in veterinary parasitology are presented in Table 2.

**Table 2:** Selected examples of molecular epidemiology studies in veterinary parasitology.

**Parasite**	**Host species**	**Molecular tool / marker**	**Geographic region**	**Main findings**	**Reference**
*Giardia duodenalis*	Dogs, cattle, humans	ITS, gdh, metabarcoding	Europe, Latin America	High assemblage diversity and zoonotic transmission patterns	Papaiakovou et al. (2023)
*Toxoplasma gondii*	Cats, wild birds, humans	Multilocus genotyping, WGS	Middle East, Europe	Three major clonal lineages and recombination hotspots	Lorenzi et al. (2016)
*Leishmania infantum*	Dogs, humans	SNPs, kDNA, WGS	Brazil, Mediterranean	Regional structuring, clonal expansion, drug-resistance variants	Carnielli et al. (2018)
*Haemonchus contortus*	Sheep, goats	SNPs, WGS	Australia, Europe	High diversity, multiple resistance haplotypes, gene flow between farms	Chaudhry et al. (2016); Frantz et al. (2023)
*Trypanosoma cruzi*	Dogs, cats, wildlife	Minicircle DNA, SNPs, WGS	South America	Distinct DTUs, domestic vs sylvatic clusters, multiple transmission events	Llewellyn et al. (2009)
*Eimeria* spp.	Poultry, ruminants	18S rRNA metabarcoding	Global	Detection of mixed-species infections in single sequencing runs	Kern et al. (2020)
*Fasciola hepatica*	Cattle, sheep	**mtDNA markers (cox1, nad1), SNP genotyping**	Europe, South America	Substructure linked to animal trade and environmental spread	Chan et al. (2021)
*Cryptosporidium* spp.	Calves, small ruminants	gp60 sequencing, WGS	North America, Europe	Identification of zoonotic subtypes and environmental persistence	Papaiakovou et al. (2023)

### Next-generation molecular tools: principles, strengths, and applications

Next generation molecular tools have profoundly transformed the field of veterinary parasitology by enabling highly sensitive, specific, and rapid detection of parasitic pathogens, including protozoa, helminths, and arthropods, across domestic and wildlife hosts (Rasool et al., 2024).These technologies enhance diagnostic capacity, support epidemiological surveillance, allow for in-depth genetic diversity analysis, and facilitate the early detection of emerging or re-emerging parasitic variants, thereby contributing to more effective prevention and control strategies (Baltrušis et al., 2020).

Unlike traditional diagnostic techniques such as microscopy, flotation, or serological assays, next-generation molecular tools have broadened the diagnostic capacity of veterinary parasitology. For instance, qPCR allows sensitive detection and quantification of parasite DNA in fecal, blood, or tissue samples, while dPCR improves precision for detecting low parasite burdens and has been applied to pathogens such as*Toxoplasma gondii*and other parasites in environmental samples (Table 3) (Li et al., 2024; Mancusi et al., 2023).

**Table 3:** Overview of next generation molecular tools used in veterinary parasitology.

**Tool**	**Principle**	**Strengths**	**Limitations**	**Veterinary applications**	**Key references**
qPCR	Real-time DNA amplification and quantification	High sensitivity and specificity; quantitative	Requires thermocycler; affected by inhibitors	Detection and quantification of parasites in clinical samples	(Li et al., 2024)
dPCR	Partitioned DNA amplification for absolute quantification	High precision; detects low loads; inhibitor tolerant	High cost; specialized equipment	Detection of *Toxoplasma gondii* and environmental parasites	(Mancusi et al., 2023)
LAMP	Isothermal amplification (60–65 °C)	Rapid; low cost; field-friendly	Limited multiplexing; contamination risk	Detection of nematodes (e.g., *Haemonchus contortus*)	(Feng et al., 2025)
RPA	Isothermal amplification (37–42 °C)	Very fast; portable; low energy	Complex primer design; higher cost/test	Detection of hemoparasites in field settings	(Feng et al., 2025)
CRISPR diagnostics	Cas-based detection with signal readout	Very high specificity; rapid	Requires pre-amplification; optimization needed	Detection of *T. gondii* and *A. marginale*	(Ma et al., 2021; Zhong et al., 2023)
NGS	High-throughput sequencing of genetic material	Detects multiple species; high resolution	Expensive; needs bioinformatics	Surveillance, diversity studies, resistance detection	(Francis et al., 2024)
qPCR	Real-time DNA amplification and quantification	High sensitivity and specificity; quantitative	Requires thermocycler; affected by inhibitors	Detection and quantification of parasites in clinical samples	(Li et al., 2024)

Similarly, isothermal amplification methods, including LAMP and RPA, provide rapid diagnostics with minimal equipment and have been used for the detection of nematodes such as*Haemonchus contortus*and other hemoparasites (Table 3) (Feng et al., 2025).

More recently, CRISPR-based diagnostics have enabled highly specific detection of pathogens such as*Toxoplasma gondii*and*Anaplasma marginale*, whereas next-generation sequencing approaches support epidemiological surveillance, biodiversity studies, and the identification of resistant parasite genotypes (Table 3) (Ma et al., 2021; Zhong et al., 2023; Francis et al., 2024).

### Advances in molecular diagnostics

Molecular diagnostic techniques have significantly expanded the capacity to detect, characterize, and monitor parasitic infections in veterinary medicine (Baltrušis & Höglund, 2023; Patiño et al., 2023). Rather than replacing classical parasitological methods, molecular approaches complement them by providing higher analytical sensitivity, improved specificity, and the ability to detect infections that would otherwise remain undiagnosed (De Cesare et al., 2024). A summary of the main molecular diagnostic methods, including their principles, advantages, limitations, and typical veterinary applications, is presented in Table 4.

**Table 4:** Overview of key molecular diagnostic methods in veterinary parasitology.

**Method**	**Principle**	**Strengths**	**Limitations**	**Applications**	**References**
qPCR / dPCR	Amplification with fluorescent probes	High sensitivity, quantification	Requires lab real-time PCR platform and technical expertise	Early detection, pathogen load estimation	(Zhong et al., 2023; Bustin, 2024)
LAMP / RPA	Isothermal amplification	Fast, portable, low cost	Lower multiplexing capacity	Field testing, resource-limited settings	(Bustin, 2024)
CRISPR-based diagnostics	Targeted cleavage guided by RNA	Ultra-specific, portable, rapid	Emerging, standardization needed	On-site detection, SNP differentiation	(Cunningham et al., 2021; Quansah et al., 2023)
NGS (metabarcoding/WGS)	High-throughput sequencing	Multiplexing, species discovery, co-infection detection	Costly, requires bioinformatics	Surveillance, genetic diversity studies	(Patiño et al., 2023)
Portable sequencing (MinION)	Nanopore real-time sequencing	Real time analysis, field deployable	Lower accuracy for single reads, coverage variability	Zoonotic surveillance, rapid outbreak tracing	(De Cesare et al., 2024; Holzschuh et al., 2024)

Modern molecular diagnostics can be classified according to their diagnostic scope, as summarized in Table 5. Targeted approaches include assays such as qPCR, dPCR, LAMP, and CRISPR-based diagnostics, which focus on detecting specific parasites or defined genetic markers. As shown in Table 5, these methods are primarily used for confirmatory diagnostics and parasite quantification because they offer high analytical accuracy and standardized laboratory protocols (Baltrušis & Höglund, 2023; Patiño et al., 2023; De Cesare et al., 2024).

**Table 5:** Classification of molecular diagnostic tools based on diagnostic scope.

**Diagnostic scope**	**Method examples**	**Target type**	**Typical use case**	**Advantages**	**References**
Targeted	qPCR, dPCR, LAMP, CRISPR	Known parasites, specific genes	Confirmatory diagnostics, quantification	High accuracy, standardized protocols	(Baltrušis & Höglund, 2023; Patiño et al., 2023; De Cesare et al., 2024)
Semi-targeted	Multiplex PCR, MLST	Multiple targets	Pathogen panels, genotyping	Broader detection, useful in outbreak investigations	(Das et al., 2023; Papaiakovou et al., 2024)
Untargeted	NGS (amplicon, metagenomics, WGS)	Broad/unknown	Mixed infections, emerging parasites, novel species detection	High sensitivity, discovery of cryptic parasites	(De Cesare et al., 2024; Naushad et al., 2025)
Real-time field surveillance	Portable nanopore sequencing (MinION)	Broad	Rapid genomic tracing, One Health surveillance	Immediate results in field	(De Cesare et al., 2024; Naushad et al., 2025)

### Molecular epidemiology and genetic diversity

Molecular epidemiology has become an essential approach in veterinary parasitology, enabling the investigation of parasite transmission, genetic diversity, and evolutionary dynamics (MacGregor et al., 2023; Rasool et al., 2024). By integrating molecular data with epidemiological information, it is possible to identify transmission routes, detect zoonotic spillovers, and monitor the emergence of resistant parasite strains (Rasool et al., 2024).

This approach is particularly relevant for parasites with complex life cycles and broad host ranges, including*Leishmania infantum*,*Toxoplasma gondii*,*Haemonchus contortus*, and*Cryptosporidium*spp., which represent important pathogens frequently investigated using molecular epidemiological tools (Carvalho et al., 2020; Galal et al., 2022; Baltrušis et al., 2022; Killander et al., 2025).

Several molecular markers are widely used in epidemiological investigations of parasitic diseases. Mitochondrial genes such as*cox1*and*nad1*are commonly applied in phylogeographic analyses and lineage tracing due to their high variability and simple amplification (Lymbery & Thompson, 2012; De Cesare et al., 2024). Ribosomal markers including ITS1, ITS2, and 18S rRNA are frequently used for species identification and metabarcoding because they are multicopy targets and allow the use of universal primers, although they may present limited resolution for closely related taxa (Preativatanyou et al., 2024).

Higher-resolution approaches such as microsatellites and SNP panels enable detailed analyses of parasite population structure and genetic diversity. These methods have been widely applied in parasites such as *Leishmania*, allowing the identification of population structuring, clonal expansion, and epidemiological patterns (Cabrera-Sosa et al., 2024a; El Mazini et al., 2023). In addition, SNP-based approaches have also contributed to the detection of genetic variants associated with drug resistance, particularly in *Leishmania infantum* (Carnielli et al., 2018).As summarized inTable 6, these molecular markers differ in their genomic targets, analytical resolution, and operational characteristics, with mitochondrial markers mainly used for lineage tracing, ribosomal genes for species identification and metabarcoding, and high-resolution markers such as microsatellites or SNP panels enabling detailed population genetic analyses.

**Table 6:** Common molecular markers and their applications in molecular epidemiology.

**Marker / approach**	**Target**	**Use cases**	**Advantages**	**Limitations**	**References**
Mitochondrial genes (cox1, nad1)	Helminths, protozoa	Phylogeography, lineage tracing	High variability, simple amplification	Maternal inheritance only	(Lymbery & Thompson, 2012; De Cesare et al., 2024)
Ribosomal DNA (ITS1, ITS2, 18S)	Protozoa, helminths	Species ID, metabarcoding	Universal Primers, multicopy	Low resolution in some taxa	(Preativatanyou et al., 2024)
Microsatellites, SNP panels	*Leishmania*, *Toxoplasma*, nematodes	Population structure, drug resistance tracking	High resolution	Requires genomic resources	(El Mazini et al., 2023; Cabrera-Sosa et al., 2024a; Carnielli et al., 2018)
WGS / Metabarcoding	Mixed parasites	Population genomics, surveillance of emerging variants	Comprehensive, high throughput	High cost, bioinformatics needed	(Rasool et al., 2024; Baltrušis et al., 2022)
MinION sequencing	Multiple taxa	Real time genomic tracing	Portable, useful for outbreak control	Lower per-read accuracy	(Patiño et al., 2023; De Cesare et al., 2024)

### Emerging and re-emerging parasitic diseases: molecular insights

Emerging and re-emerging parasitic diseases represent a major challenge for animal health, food security, and public health, particularly within the One Health framework. These infections often arise at the interface between livestock, wildlife, humans, and the environment, where environmental drivers such as climate change, land-use modification, animal movement, and trade influence parasite distribution and genetic diversity (Caminade et al., 2019 Doyle et al., 2022) .

In this context, emerging parasitic diseases are defined as infections that have newly appeared in a population or are rapidly increasing in incidence, geographic distribution, or host range. In contrast, re-emerging diseases refer to previously known infections that were under control but are now resurging due to ecological, environmental, or anthropogenic changes.

Molecular tools have transformed the detection and interpretation of these events. Techniques such as qPCR, dPCR, metabarcoding, and whole genome sequencing enable early detection of parasite circulation, identification of resistance associated variants, and discrimination of closely related lineages (Wang et al., 2024).

It is important to note that some parasites presented in Table 7 (e.g., *Giardia* spp., *Leishmania* spp., and *Haemonchus* contortus) are well-established pathogens and are not strictly classified as emerging or re-emerging. In this review, they are included as representative models to illustrate how molecular tools can detect epidemiological shifts, such as changes in transmission dynamics, distribution, or resistance patterns (Kotze & Prichard, 2016; Galal et al., 2019).

**Table 7:** Actionable molecular insights for emerging/re-emerging parasites.

**Insight type**	**Molecular signal (examples)**	**Typical assay**	**What it tells you (decision supported)**	**Representative parasite contexts**	**References**
Early detection & cryptic circulation	Low-copy DNA/RNA; multi-species reads	qPCR/dPCR; metabarcoding; metagenomics	Enables early detection before clinical peaks	*Cryptosporidium*in contaminated calf environments;*Eimeria*in subclinical poultry flocks	(Zhong et al., 2023)
Source attribution & spillover (One Health)	SNP clustering; gp60 subtypes; host-switch signals	WGS/SNP panels; gp60 typing	Identifies reservoirs and zoonotic transmission routes	*Trypanosoma cruzi* across wildlife–domestic cycles; zoonotic*Cryptosporidium parvum*;*Leishmania infantum*in dogs	(Caminade et al., 2019; Doyle et al., 2022)
Velocity of spread	Low diversity; short SNP distances	Phylogenies; haplotype networks	Quantifies transmission dynamics and spread	*Leishmania*outbreaks in endemic areas;*Babesia* spread via tick expansion	(Caminade et al., 2019; Doyle et al., 2022)
Drug/anthelmintic resistance	Resistance SNPs; selective sweeps	Targeted SNPs; WGS	Identifies resistance hotspots for treatment decisions	Anthelmintic-resistant*Haemonchus contortus*in small ruminants	(Kotze & Prichard, 2016)
Recombination/novel variants	Mosaic genomes; new alleles	WGS; amplicon phasing	Detects emerging variants requiring surveillance	Atypical*Toxoplasma gondii*genotypes linked to increased virulence	(Galal et al., 2019; Zhong et al., 2023)
Environmental persistence & re-introduction	Recurrent detection in environmental samples	qPCR/dPCR; nanopore	Indicates environmental reservoirs and reinfection risk	Waterborne*Cryptosporidium*and*Giardia*contamination	(Zhong et al., 2023)

### Integration of molecular and conventional diagnostic approaches

While molecular tools have transformed veterinary parasitology, conventional parasitological methods remain essential for routine diagnosis and field surveillance. Techniques such as flotation, sedimentation, blood smears, and fecal egg counts continue to provide rapid, low-cost diagnostic information and remain widely used in veterinary laboratories and field investigations (Kotze et al., 2014; Ryan et al., 2019).

As summarized inTable 8, integrated diagnostic workflows can be applied across different parasitological contexts. For example, gastrointestinal parasites may be initially detected by microscopy-based fecal examination and subsequently confirmed using molecular assays such as qPCR or metabarcoding. Similarly, blood-borne protozoa can be visualized in blood smears, while molecular tools increase diagnostic sensitivity and allow accurate species identification. Combined strategies are also applied in environmental surveillance of waterborne parasites such as*Cryptosporidium*and*Giardia*, as well as in monitoring anthelmintic resistance by linking fecal egg count results with molecular markers (Ryan et al., 2019; Doyle et al., 2022).

**Table 8:** Integration of conventional and molecular diagnostics in veterinary parasitology.

**Diagnostic target**	**Conventional method**	**Molecular method**	**Integration strategy**	**Benefits**	**Representative parasite contexts**
Gastrointestinal parasites	Flotation, sedimentation, microscopy	qPCR, LAMP, metabarcoding	Screening by microscopy; confirmation and species-level identification by molecular tools	Rapid screening + accurate species/genotype information	*Haemonchus contortus, Eimeria* spp.
Blood protozoa	Blood smears	qPCR, dPCR, CRISPR assays	Initial visualization and parasitemia estimation, followed by molecular confirmation	Combines sensitivity and field applicability	*Babesia* spp., *Trypanosoma* spp*.*
Zoonotic waterborne parasites	Microscopy, immunofluorescence	qPCR/dPCR, metabarcoding, MinION	Environmental screening with microscopy; molecular typing for source attribution	Early detection of cryptic transmission and reservoirs	*Cryptosporidium, Giardia*
Drug resistance monitoring	Fecal egg counts	SNP genotyping, WGS	Phenotypic screening with molecular confirmation	Links phenotypic resistance with genetic markers	*Haemonchus contortus*

### Surveillance and One Health applications

The integration of molecular diagnostics with One Health surveillance has strengthened the detection and monitoring of parasitic diseases at the animal–human–environment interface. These approaches allow the identification of silent transmission, early detection of infections, and genetic characterization of parasites, supporting outbreak investigation and zoonotic risk assessment (Ryan et al., 2019).

As summarized inTable 9, different molecular tools support specific surveillance objectives. For instance, qPCR and dPCR enable highly sensitive early detection of parasites such as*Cryptosporidium*spp. and*Giardia*spp., while portable sequencing technologies such as Nanoporeallow real-time genomic tracing in field conditions (Holzschuh et al., 2024).

**Table 9:** Molecular surveillance applications, highlighting their purpose, tools used, benefits, and representative parasites.

**Surveillance Objective**	**Molecular Tools**	**Key Advantages**	**Representative Parasites**	**References**
Early detection in hosts and environment	qPCR, dPCR, metabarcoding	High sensitivity; detects low-intensity infections; reveals silent transmission	*Cryptosporidium* spp.,*Giardia* spp.	(Papaiakovou et al., 2023)
Real-time genomic tracing in the field	Nanopore sequencing (MinION)	Rapid genomic data generation; enables on-site decisions	Parasites in field conditions	(Holzschuh et al., 2024)
Transmission pathway reconstruction	WGS, MLST, SNP analysis	High-resolution tracking of transmission routes and reservoirs	Zoonotic parasites	(Bruno et al., 2024)
Genomic epidemiology in endemic regions	WGS, population genomics	Characterizes genetic diversity and host–parasite dynamics	*Leishmania infantum*	(Cabrera-Sosa et al., 2024a)
One Health surveillance and risk assessment	Integration of molecular + epidemiological data	Supports outbreak investigation and zoonotic risk assessment	Multi-host parasitic systems	(Papaiakovou et al., 2023)

In addition, high-resolution approaches including whole-genome sequencing, multilocus sequence typing, and single-nucleotide polymorphism analyses help reconstruct transmission pathways and identify parasite reservoirs, improving the understanding of epidemiological dynamics and the spread of zoonotic infections (Bruno et al., 2024).

These genomic tools have been increasingly applied to parasites such as*Leishmania infantum*to investigate genetic diversity, transmission patterns, and host reservoirs in endemic regions (Kuhls et al., 2011) .

### Applications for domestic animals and livestock

Wildlife species are essential components of parasitic disease ecology and act as sentinel hosts in One Health surveillance systems (Thompson & Ash, 2016). Because they occur at the interface between natural ecosystems, livestock production systems, and human populations, wildlife may function as reservoirs, amplifiers, or sources of spillover for several zoonotic and veterinary parasites (Babayani et al., 2022). Traditional diagnostic methods such as microscopy remain widely used but often present limited sensitivity for detecting low parasite burdens or subclinical infections in free-ranging wildlife populations (Ryan et al., 2019).

Modern molecular platforms such as qPCR, metabarcoding, multilocus sequence typing (MLST), and WGS have expanded the capacity for wildlife surveillance, frequently using non-invasive samples such as feces or environmental material (Bohmann et al., 2014). These tools enable the detection of parasite circulation in wildlife populations before outbreaks occur in domestic animals or humans. For example, metabarcoding and ITS-based sequencing have revealed diverse gastrointestinal nematode communities in wild ruminants, including species shared with livestock such as*Haemonchus contortus*(Avramenko et al., 2017).

Similarly, molecular surveillance has improved the detection of zoonotic parasites including*Toxoplasma gondii*,*Cryptosporidium* spp.,*Leishmania* spp*.*, and*Trypanosoma* spp*.*in wildlife hosts such as carnivores, bats, rodents, birds, and non-human primates (Aderibigbe et al., 2015). As illustrated inTable 10, molecular tools are applied across multiple wildlife host groups. For example, qPCR, MLST, and WGS are used for genotyping*Toxoplasma gondii*in wild carnivores, ITS-based metabarcoding enables the detection of mixed gastrointestinal nematode infections in wild ruminants, and MinION sequencing and metabarcoding facilitate the identification of protozoan parasites such as*Plasmodium*and*Trypanosoma*in non-human primates. In addition, sequencing of markers such as gp60 combined with qPCR is used to monitor waterborne zoonotic parasites such as*Cryptosporidium*and*Giardia*in wild birds and aquatic ecosystems.

**Table 10:** Molecular tools applied to the detection and surveillance of zoonotic parasites in wildlife and free-ranging animal populations.

**Wildlife host / ecosystem**	**Parasite(s)**	**Molecular tool**	**Application and relevance**	**References**
Wild canids, foxes, wild felids	*Toxoplasma gondii*	qPCR, MLST, WGS	Genotyping of strains; identifying wildlife reservoirs; understanding spillover dynamics	(Lorenzi et al., 2016; Fatemi et al., 2019)
Wild ruminants (deer, antelope, boar)	GI nematodes (*Haemonchus contortus, Eimeria* spp.)	ITS-2 metabarcoding, WGS	Detection of mixed infections and resistant genotypes in free-ranging populations	(Avramenko et al., 2017)
Non-human primates	*Plasmodium* spp., *Trypanosoma cruzi*	qPCR, metabarcoding, MinION	Detection of zoonotic parasites with sylvatic cycles; early warning for spillover	(Aderibigbe et al., 2015)
Bats, rodents	*Leishmania* spp., *Trypanosoma* spp.	WGS, MLST	Identifying cryptic reservoirs and phylogenetic lineages	(Aderibigbe et al., 2015)
Wild birds, waterfowl	*Cryptosporidium* spp., *Giardia* spp.	gp60 sequencing, qPCR, metabarcoding	Environmental surveillance; zoonotic transmission via water sources	(Berghella & Di Mascio, 2020)

### Technological innovations and bioinformatics tools

Technological innovations are shifting veterinary parasitology toward faster, more automated, and predictive surveillance approaches. Recent advances emphasize portability, scalable bioinformatics analysis, and the integration of genomic and environmental data streams to support real-time epidemiological decision-making (Holzschuh et al., 2024; Monteiro et al., 2025).

Portable sequencing technologies have become an important component of modern parasite surveillance. Platforms such as Oxford Nanopore’s MinION enable real-time genomic sequencing directly in the field, facilitating rapid pathogen identification during outbreak investigations and supporting surveillance in remote or resource-limited environments (Holzschuh et al., 2024). In parallel, automated bioinformatics workflow systems including Galaxy, Nextflow, and Snakemake enable reproducible and scalable pipelines for the analysis of genomic and metabarcoding datasets, improving the efficiency and reproducibility of large-scale molecular analyses (Di Tommaso et al., 2017; Afgan et al., 2018).

Recent advances also include the integration of artificial intelligence and machine learning algorithms for lineage classification, detection of resistance-associated genetic markers, and prediction of parasite transmission dynamics across space and time (Parija & Poddar, 2024; Monteiro et al., 2025). In addition, open genomic databases such as EuPathDB, WormBase ParaSite, and GenBank provide curated genomic resources that support comparative genomics and phylogenetic analyses of parasitic organisms (Warrenfeltz et al., 2018).

As illustrated in Table 11, several digital and molecular platforms are currently being integrated to strengthen parasite surveillance systems. For example, portable sequencing platforms such as MinION enable rapid genomic characterization of parasites during field investigations, while bioinformatics workflow managers such as Galaxy and Nextflow facilitate large-scale genomic data analysis. In addition, machine learning tools assist in predicting parasite transmission patterns and detecting resistance markers, whereas open-access genomic repositories provide reference datasets for comparative genomic studies. Finally, geospatial platforms such as QGIS and ArcGIS enable the integration of molecular, ecological, and epidemiological data to map parasite distribution and identify areas of increased transmission risk within the One Health framework.

**Table 11:** Technological and bioinformatics tools drive innovation in veterinary parasitology.

**Category**	**Tool / Platform**	**Application**	**Relevance to parasite surveillance**	**References**
Portable sequencing	MinION (Oxford Nanopore)	Real-time genomic analysis in the field	Rapid outbreak response, low-resource settings	(Holzschuh et al., 2024)
Workflow automation	Galaxy, Nextflow, Snakemake	Reproducible and scalable bioinformatics pipelines for WGS, metabarcoding, and MLST	High-throughput processing	(Di Tommaso et al., 2017; Afgan et al., 2018)
AI and machine learning	ML algorithms, predictive modeling tools	Lineage classification, resistance marker detection, spatiotemporal transmission modeling	Predictive surveillance	(Parija & Poddar, 2024; Monteiro et al., 2025)
Open genomic databases	EuPathDB, WormBase ParaSite, GenBank	Access to curated parasite genomes, comparative analyses, phylogenetics	Data integration and reference support	(Warrenfeltz et al., 2018)
Geospatial integration	QGIS, ArcGIS	Linking molecular and ecological data for mapping and risk modeling	One Health early warning systems	(Holzschuh et al., 2024; Monteiro et al., 2025)

### CRISPR-based diagnostics and lab-on-a-chip platforms

Recent advances in CRISPR based diagnostics and microfluidic lab on chip technologies have expanded the molecular detection capacity for parasitic diseases by enabling rapid, highly specific, and portable diagnostic platforms (Zhong et al., 2023). CRISPR Cas systems such as SHERLOCK (Cas13) and DETECTR (Cas12) detect nucleic acids through programmable guide RNAs that activate collateral cleavage of reporter molecules after target recognition, allowing sensitive detection of parasite DNA or RNA (Gootenberg et al., 2017; Zhong et al., 2023). These systems are frequently combined with isothermal amplification methods such as RPA or LAMP, which improve analytical sensitivity and enable rapid detection without conventional thermocyclers (Sun et al., 2024).

In parallel, microfluidic lab on chip platforms integrate sample preparation, nucleic acid amplification, and signal detection within compact devices, reducing contamination risks and supporting faster diagnostic workflows (Meng et al., 2025). These technologies have been applied to the detection of parasites such as*Toxoplasma gondii*, *Leishmania* spp., *Trypanosoma cruzi*, and *Anaplasma marginale*, supporting surveillance in livestock, wildlife, and companion animals (Zhong et al., 2023; Sun et al., 2024).

As illustrated inTable 12, several CRISPR based diagnostic configurations are currently used in parasite detection, including SHERLOCK and DETECTR fluorescence assays, CRISPR, LAMP microfluidic platforms, portable RPA CRISPR systems, and lateral flow CRISPR strips for visual detection, which enable rapid molecular diagnostics in laboratory and field settings (Liang et al., 2024).

**Table 12:** CRISPR-based and lab-on-a-chip diagnostic platforms applied to veterinary parasitology and One Health surveillance.

**Platform / Technology**	**Detection principle**	**Target type**	**Readout mode**	**Advantages**	**Limitations**	**Reported applications**	**References**	
SHERLOCK (Cas13)	Collateral cleavage upon RNA recognition	RNA/DNA	Fluorescence, lateral flow	Ultra-specific, rapid, portable, minimal equipment	Requires RPA pre-amplification, RNA handling	*Toxoplasma gondii*, *Leishmania* spp.		(Gootenberg et al., 2017)
DETECTR (Cas12)	DNA recognition and reporter cleavage	DNA	Fluorescence, lateral flow	High specificity, LAMP compatible, rapid detection (<1 h)	Primer optimization critical	*Leishmania* spp., *Trypanosoma cruzi*, *Anaplasma marginale*		(Zhong et al., 2023)
CRISPR + LAMP microfluidics	Isothermal amplification + Cas12 detection integrated on chip	DNA	On-chip fluorescence, digital	Integrated workflow, low contamination, fast turnaround	Fabrication cost, moderate complexity	*Toxoplasma gondii*, helminths		(Meng et al., 2025)
RPA + CRISPR lab-on-a-chip	RPA amplification + CRISPR detection on portable chip	DNA/RNA	Fluorescence, digital	Portable, low-cost, field deployable, suitable for remote regions	Limited multiplexing	Protozoa and helminths in livestock and wildlife		(Sun et al., 2024)
Lateral flow CRISPR strips	Cas12/13 + lateral flow detection strip	DNA/RNA	Colorimetric (visual)	Easy interpretation, no specialized equipment, suitable for low-resource settings	Lower sensitivity than fluorescence	*Leishmania infantum*, *Toxoplasma gondii*, *Eimeria* spp.		(Zhong et al., 2023; Liang et al., 2024)

### Environmental DNA (eDNA) and One Health surveillance

Environmental DNA approaches have become an important strategy for surveillance of parasitic diseases within the One Health framework because they allow detection of parasite genetic material in environmental matrices without directly sampling hosts or vectors (Sengupta et al., 2022). For example, surface water and effluent samples can be analyzed for parasites such as *Cryptosporidium*, *Giardia*, helminths, and *Toxoplasma* using qPCR, digital PCR, or metabarcoding, helping identify contamination sources and guide sanitation measures (Sengupta et al., 2022). Similarly, soil, sediment, and manure samples may contain parasite eggs or oocysts such as Eimeria and Toxoplasma, which can be detected using targeted PCR or metabarcoding to evaluate environmental persistence (Moreno et al., 2024; Piccirillo et al., 2024).

As illustrated inTable 13, additional matrices can also support parasite surveillance. Biofilms and trough slime may accumulate protozoan DNA or helminth stages, while dust or air filters may contain aerosolized parasite stages or vector DNA, enabling environmental monitoring in animal facilities (Mikko et al., 2025; Vassallo*,* 2025). In addition, vectors and invertebrates can be screened for parasite DNA present in blood meals, and food or fomites may contain parasite DNA such as *Toxoplasma* or *Cryptosporidium*, supporting risk assessment and food safety monitoring (Sengupta et al., 2022; Piccirillo et al., 2024).

**Table 13:** eDNA workflows for veterinary–One Health surveillance: matrices, assays, decisions, and quality control.

**Matrix & context**	**Typical targets (examples)**	**Preferred assays (by objective)**	**Decision-use (examples)**	**Key advantages**	**Main limitations & QC essentials**	**Key references**
Surface water / effluent	*Cryptosporidium*(gp60),*Giardia*(gdh/tpi), helminths (18S/ITS),*Toxoplasma*	qPCR/dPCR (early warning); metabarcoding (community); nanopore (rapid field)	Identify contamination sources; guide fencing, water sanitation	Non-invasive; integrates upstream signals	Inhibitors, dilution; use blanks, IPC, volume normalization	(Sengupta et al., 2022)
Soil / sediment / manure	Eggs/oocysts (*Eimeria*,*Toxoplasma*), ITS-2 nematodes	qPCR/dPCR (targeted); metabarcoding (pasture burden)	Manure management, grazing rotation	Captures environmental persistence	Heterogeneous DNA; compositing, inhibition checks	(Moreno et al., 2024; Piccirillo et al., 2024)
Biofilms & trough slime	Biofilm protozoa, resistant helminth DNA	qPCR/dPCR, shotgun metagenomics (AMR context)	Sanitation SOPs, water point design	DNA enrichment, stable signal	Biofilm inhibitors; time-series, blanks	(Mikko et al., 2025)
Dust / air filters	Aerosolized oocysts/eggs; vector DNA	qPCR for targets; metabarcoding for aero-biome	Improve ventilation, PPE, cleaning	Non-intrusive	Low template; high-volume sampling	(Vassallo, 2025)
Vectors & invertebrate samplers	Parasite DNA in blood meals	Amplicon metabarcoding; qPCR	Map host–parasite networks	Extends surveillance to wildlife	Background DNA; blocking primers	(Sengupta et al., 2022)
Food & fomites	*Toxoplasma*,*Cryptosporidium*, GI helminths	qPCR/dPCR (hazard detection); LAMP/CRISPR POC	Batch holds, hygiene verification	Direct link to food safety	Matrix inhibition; recovery spikes	(Piccirillo et al., 2024)

### Molecular pathogenesis and host–parasite interactions

Genetic diversity plays a critical role in parasite infectivity, host immune modulation, and disease progression, highlighting the importance of molecular mechanisms in host–parasite interactions (Silvestrini et al., 2024).Many parasites rely on specialized molecular factors that mediate host cell invasion and immune modulation. For example,*Toxoplasma gondii* secretes rhoptry and dense granule proteins such as ROPs and GRAs, which modulate innate signaling pathways, inhibit apoptosis, and promote intracellular niche formation while activating host IFN-γ pathways and inflammasome responses (Wang et al., 2020; Kim et al., 2020; Yoon et al., 2024).

Similarly,*Leishmania* species express virulence molecules including the metalloprotease GP63 and the surface lipophosphoglycan (LPG), which interfere with complement activation and macrophage signaling pathways, influencing Th1/Th2 polarization and disease severity (Brittingham & Mosser, 1996; Forestier et al., 2015). In apicomplexan parasites such as*Eimeria spp*., invasion of intestinal epithelial cells depends on apical complex proteins and microneme adhesins, which trigger epithelial inflammation and IL-1β responses during infection (Jiang et al., 2020).

In addition,*Cryptosporidium parvum* uses the gp60 glycoprotein and mucin like surface proteins to adhere to intestinal epithelial cells and invade enterocytes, activating mucosal innate immune responses including interferon signaling pathways (Coelho et al., 2023; Hayes et al., 2023). As illustrated in Table 14, these host parasite interactions involve distinct virulence factors, immune pathways, and molecular markers that are increasingly used in diagnostics, strain typing, and epidemiological surveillance.

**Table 14:** Key molecular mechanisms of host–parasite interactions.

**Parasite**	**Key molecular factors**	**Mechanism of interaction**	**Host immune response**	**Diagnostic / epidemiological relevance**	**Recent references**
*Toxoplasma gondii*	Rhoptry proteins (ROPs), dense granule proteins (GRAs), MICs	Modulation of innate signaling pathways, evasion of apoptosis, intracellular niche formation	Activation of IFN-γ pathways, inflammasome engagement, modulation of host gene expression	ROP and GRA polymorphisms support strain typing and virulence prediction; key vaccine targets	(Kim et al., 2020; Yoon et al., 2024; Silvestrini et al., 2024)
*Leishmania* spp.	GP63 metalloprotease, lipophosphoglycan (LPG), HSP70, LRV2 (endosymbiont virus)	Cleavage of host signaling proteins, complement evasion, macrophage modulation, viral enhancement of virulence	Th1/Th2 polarization, suppression of NF-κB and NO response	GP63 and LPG are widely used in immunodiagnostic development; LRV2 associated with severe disease forms	(Brittingham & Mosser, 1996; Forestier et al., 2015)
*Eimeria* spp.	Apical complex proteins, microneme adhesins	Invasion of intestinal epithelial cells via apical complex	IL-1β response, epithelial barrier damage, local inflammation	Microneme proteins are promising vaccine targets and molecular markers	(Jiang et al., 2020)
*Cryptosporidium parvum*	GP60 glycoprotein, mucin-like surface proteins	Adhesion to intestinal epithelial cells, invasion of enterocytes	IFN-λ signaling, mucosal innate activation	gp60 subtyping remains standard in outbreak tracking and molecular surveillance	(Coelho et al., 2023; Hayes et al., 2023)

### High-throughput platforms and point-of-care technologies

High-throughput (HT) and point-of-care (POC) molecular systems are reshaping veterinary parasitology by accelerating time to result, expanding surveillance capacity, and extending diagnostics beyond centralized laboratories (Ryan et al., 2019). HT platforms such as automated qPCR arrays, digital PCR (dPCR), targeted amplicon sequencing, and shotgun or WGS enable high-volume testing with high analytical sensitivity and standardized workflows, supporting large-scale prevalence studies, parasite genotyping, and monitoring of drug-resistance markers (Jiang et al., 2020).

As summarized inTable 15, multiplex qPCR and dPCR platforms can process large numbers of samples using high-density well arrays or droplet-based systems, providing precise quantification and reproducible laboratory workflows, although these approaches may involve higher costs and limited discovery of novel taxa. In contrast, amplicon or metagenomic NGS approaches, including ITS or 18S metabarcoding and WGS, allow the detection of mixed infections, identification of novel variants, and detailed genotyping of parasite populations, but typically require advanced bioinformatics infrastructure and greater financial investment (Rathinasamy et al., 2018; Chiu & Miller, 2019).

**Table 15:** High throughput (HT) and point of care (POC) molecular technologies.

**Modality**	**Example platform**	**Key strengths**	**Limitations**	**Field readiness**	**Representative applications / References**
Multiplex qPCR / dPCR (HT)	96–384 well arrays; droplet dPCR	High sensitivity, quantification, standardized workflows	High cost; limited discovery of novel taxa	Laboratory	(Jiang et al., 2020)
Amplicon / metagenomic NGS (HT)	18S/ITS metabarcoding; WGS	Detects mixed infections and novel variants; supports genotyping	Requires bioinformatics and higher cost	Laboratory	(Rathinasamy et al., 2018; Chiu & Miller, 2019)
Portable nanopore sequencing (POC +)	MinION / Flongle	Real-time genomic tracing, deployable in field	Accuracy variability, QC needed	Field deployable	(Quick et al., 2016)
Isothermal amplification (POC)	LAMP / RPA	Rapid (< 40 min), low equipment needs	Contamination risk if open tube	Field ready	(Boonbanjong et al., 2022)
CRISPR-based assays (POC)	Cas12/Cas13 + LAMP or RPA	Ultra-specific, visual or fluorescent readout	Requires pre-amplification	Field ready	(Bokulich et al., 2018; Bolyen et al., 2019)

In parallel, POC technologies are expanding diagnostic capacity outside traditional laboratories. As illustrated inTable 16, isothermal amplification methods such as loop-mediated LAMP and recombinase RPA enable rapid detection within minutes using minimal equipment, making them particularly suitable for field diagnostics and low-resource settings (Boonbanjong et al., 2022),.

**Table 16:** Examples of data integration, AI, and bioinformatics pipelines used in veterinary parasitology.

**Application**	**Tool / Platform**	**Key functions**	**Advantages**	**Limitations**	**Representative studies**
Sequence quality control and processing	QIIME 2, USEARCH, fastp	Quality filtering, trimming, clustering, taxonomic assignment	Widely used, reproducible workflows	Requires bioinformatics expertise	(Jiang et al., 2020)
Taxonomic classification and community profiling	Kraken2, MetaPhlAn	Rapid classification of reads, metagenomic profiling	High speed, compatible with large datasets	Sensitive to database quality	(Rathinasamy et al., 2018; Chiu & Miller, 2019)
Multiomics data integration	Galaxy, Nextflow	Combines genomic and environmental metadata	Modular, cloud compatible	Requires structured data	(Boonbanjong et al., 2022)
AI-assisted parasite detection	TensorFlow, PyTorch	Pattern recognition, image-based diagnosis	Automation, scalable	Needs training datasets	(Bokulich et al., 2018)
Outbreak prediction and surveillance	EpiForecast, R Shiny	Spatiotemporal modeling, forecasting	Real time dashboards, decision support	Model uncertainty	(Quick et al., 2016)
Database and reference management	NCBI, EuPathDB	Curated reference sequences and metadata	Standardized, global	Update lag in some taxa	(Chiu & Miller, 2019)

Emerging CRISPR-based assays using Cas12 or Cas13 combined with isothermal amplification further improve specificity and allow visual or fluorescent readouts for rapid pathogen detection (Bokulich et al., 2018). In addition, portable nanopore sequencing platforms, such as MinION or Flongle, allow real-time genomic analysis directly in the field, facilitating outbreak investigations and genomic surveillance despite current limitations related to sequencing accuracy and quality control requirements (Quick et al., 2016).

Together, HT platforms provide depth, throughput, and analytical precision, whereas POC tools offer speed, portability, and accessibility for diagnostics at farms, clinics, and wildlife surveillance sites. This complementary and tiered diagnostic framework strengthens both immediate clinical decision-making and long-term genomic surveillance strategies within integrated One Health monitoring systems (Ryan et al., 2019).

### Data integration, AI, and bioinformatics pipelines

The growing volume of molecular data generated in veterinary parasitology requires robust bioinformatics pipelines capable of transforming complex datasets into biologically meaningful information (Afgan et al., 2018; Bolyen et al., 2019). The integration of multi-omics data, including genomic and epidemiological information, has strengthened diagnostic interpretation, parasite surveillance, and the early detection of emerging parasitic diseases (Ryan et al*.,* 2019).

As summarized inTable 17, bioinformatics pipelines support multiple analytical steps, including sequence quality control, taxonomic classification, and data integration. Tools such as QIIME2, USEARCH, and fastp are commonly used for sequence filtering and taxonomic assignment in metabarcoding studies, while classification algorithms such as Kraken2 and MetaPhlAn enable rapid taxonomic prof of large sequencing datasets (Bokulich et al., 2018).

**Table 17:** Selected examples of molecular surveillance of parasitic diseases by region.

**Region**	**Parasites / Pathogens**	**Molecular tool**	**Reported prevalence**	**Representative studies**
North America & Europe	*Leishmania infantum* (dogs), others	PCR, qPCR, sequencing	Substantial dog infection burdens in Mediterranean foci; molecular confirmation recommended	(Symeonidou et al., 2023)
Asia	*Cryptosporidium, Eimeria, Leishmania*	PCR, NGS	Variable livestock/companion-animal detection (program dependent)	(Cabrera-Sosa et al., 2024a)
Africa	*Trypanosoma*	PCR, portable sequencing	Endemic burdens tracked by molecular tools in control programs	(Nakayima et al., 2012; Hounyèmè et al., 2022)
Latin America (regional)	*Mansonella ozzardi*; malaria markers	PCR, targeted sequencing	Community infection >30% in some Amazonian villages; high pfhrp2/3 deletion frequency in Peru	(Montoya-Alonso et al., 2020)
Brazil (South)	*Leishmania infantum* (dogs, vectors)	qPCR, ITS sequencing	Dogs: ~4.2% by conjunctival swab; vector infection detected in Nyssomyia neivai	(Dahmer et al., 2023; Cabrera-Sosa et al., 2024a)
Brazil (South)	*Leishmania* spp. in bats	PCR	Bats: ~2.7% positive in RS survey	(Cardoso et al., 2024)
Brazil (North)	*Leishmania* spp. (human cases)	PCR, sequencing	Positivity ≈49% among suspected cases; multiple species circulating	(Ratzlaff et al., 2022)

In addition, workflow platforms such as Galaxy and Nextflow facilitate the integration of genomic and epidemiological data, while AI and machine learning frameworks, including TensorFlow and PyTorch, support automated parasite detection and predictive modeling. Together with reference databases such as NCBI and EuPathDB, these tools form integrated analytical infrastructures that support sequence processing, data integration, and AI-assisted diagnostics in veterinary parasitology, as illustrated inTable 17.

### Global and Brazilian perspectives on molecular advances in veterinary parasitology

Molecular tools have reshaped parasite surveillance worldwide, enabling sensitive detection, high-resolution genotyping, and faster outbreak investigation across domestic animals, wildlife. Countries in North America and Europe have incorporated multiplex qPCR, digital PCR, metabarcoding, nanopore sequencing, and WGS into national and regional programs, supporting risk-based monitoring and targeted control. For instance, canine *Leishmania infantum* remains a major focus in the Mediterranean basin, where molecular and serologic surveys routinely document substantial dog infection burdens in endemic areas, underscoring the value of molecular confirmation for mapping transmission risk (Symeonidou et al., 2023).

In Asia, large-scale molecular surveillance is used for *Leishmania donovani*, *Cryptosporidium*, *Eimeria*, and other parasites in livestock and companion animals, while malaria programs have adopted genetic markers and sequencing to evaluate diagnostic performance and transmission dynamics. In Peru’s Loreto region, pfhrp2/3 gene deletions in *Plasmodium falciparum* a critical issue for HRP2-based rapid tests have been measured at very high frequencies in molecular surveys, emphasizing how genomics directly informs test selection and policy (Cabrera-Sosa et al., 2024a).

Across Africa, molecular epidemiology is central for *Trypanosoma* and *Plasmodium* surveillance, with PCR and portable sequencing increasingly used to quantify endemic burdens and track parasite populations for control programs. Oceania (Australia and New Zealand) likewise applies molecular diagnostics to wildlife and production animals (e.g., *Toxoplasma*, *Cryptosporidium*), feeding genomic data into global reference resources to support lineage tracing and comparative risk assessments (Nakayima et al., 2012; Montoya-Alonso et al., 2020; Hounyèmè et al., 2022).

In Latin America, adoption is expanding but heterogeneous. Studies from the Colombian Amazon using molecular diagnostics revealed high community infection burdens of *Mansonella ozzardi*, and regional malaria programs are leveraging genomic surveillance to address RDT failure risks due to pfhrp2/3 deletions.

These examples illustrate the promise of molecular tools for improving parasite surveillance and diagnostic accuracy across the region. However, they also highlight persistent implementation gaps that still limit the establishment of consistent and standardized molecular surveillance systems across countries (Dahmer et al., 2023).

Brazil stands out as a regional leader with extensive academic and reference-lab activity and growing translation into public veterinary health. Recent Brazilian studies document (i) molecular detection of *L. infantum* in dogs (≈4.2% by conjunctival swab) alongside infected phlebotomines in the South, even where the primary vector (*Lutzomyia longipalpis*) has not been detected; (ii) *Leishmania* DNA in bats (~2.7% in surveyed cohorts) in Rio Grande do Sul; and (iii) high species diversity of *Leishmania* in the North (Roraima), with nearly half of clinically suspected cases testing positive across parasitological/molecular methods.

Together, these data highlight the importance of integrating animal, vector, and human testing with molecular confirmation to guide control strategies. Table 18 consolidates representative regional examples and their molecular tools, with links to the underlying studies (Almeida et al., 2021; Ratzlaff et al., 2022).

**Table 18:** Policy and regulatory frameworks supporting molecular surveillance with journal examples.

**Region / Organization**	**Parasite / Focus**	**Molecular or Policy Approach**	**Application**	**Journal**	**Year**
Global (WOAH context)	*Leishmania* spp.	Integration of molecular diagnostics into global surveillance	Standardization and coordination	Transboundary and Emerging Diseases	2023
Global (FAO context)	AMR in parasites	Genomics and AMR integration in One Health	Global surveillance framework	One Health	2023
Global (WHO context)	Zoonotic parasites	Molecular tools for early warning	Coordinated surveillance	The Lancet Global Health	2022
Europe (EFSA)	*Echinococcus multilocularis*	Molecular reference lab networks	Standardized surveillance	Eurosurveillance	2022
USA (USDA NAHLN)	*Trichinella* spp.	National molecular surveillance	Veterinary programs	Journal of Veterinary Diagnostic Investigation	2023
Peru	*Plasmodium falciparum*	pfhrp2/3 genomic surveillance	Diagnostic policy	Scientific Reports	2024
Brazil	*Leishmania infantum*	qPCR in dogs and vectors	Control strategies	Parasites & Vectors	2024
Brazil	*Leishmania* spp. (bats)	Wildlife surveillance	One Health integration	Parasitology Research	2022
Brazil	*Toxoplasma gondii*	Molecular surveillance in livestock	Food safety	Veterinary Parasitology	2020

### Limitations of the present review

While this review offers a comprehensive overview of advances in the application of molecular tools in veterinary parasitology, certain limitations must be acknowledged. The literature search was restricted to four major databases (PubMed, Scopus, Web of Science, and SciELO) and to publications written in English, Portuguese, or Spanish. Consequently, relevant studies published in other languages or indexed in additional regional or specialized databases may not have been captured.

The time frame considered (2000–2025) may also have excluded older but still relevant foundational studies. Furthermore, because this work is a narrative review, no formal quality assessment or meta-analysis was performed, which may introduce a degree of subjectivity into the interpretation of results. The considerable heterogeneity among the available studies, including variations in study design, host species, target parasites, and molecular methodologies, posed challenges to direct comparison across research findings.

Although this review incorporates data from multiple regions around the world, the depth and availability of research are not uniform across all geographic areas. Some regions, including Latin America, where Brazil stands out due to the volume of available studies, as well as parts of Europe, Asia, and North America, have been explored in greater detail, while other regions remain comparatively underrepresented in the literature. Despite these constraints, this review integrates global evidence with regional insights and provides valuable perspectives on the current state, practical applications, and future directions of molecular approaches in veterinary parasitology.

### Policy, regulation, and future perspectives

The integration of molecular diagnostics into veterinary parasitology has expanded rapidly over the past decade, but this progress has not always been matched by regulatory and policy development. To ensure reliability, reproducibility, and international comparability of results, molecular tools must be embedded within well-structured legal and policy frameworks that address assay validation, biosafety, quality assurance, and data interoperability.

At the global level, organizations such as the World Organization for Animal Health (WOAH), the Food and Agriculture Organization (FAO), and the World Health Organization (WHO) have emphasized the incorporation of molecular and genomic data into coordinated surveillance strategies (Catalano et al., 2024).These frameworks highlight the role of molecular tools in strengthening early warning systems, harmonizing diagnostics, and improving zoonotic disease response capacity.

Europe and the United States provide some of the clearest operational examples of how molecular surveillance can be institutionalized. The European Food Safety Authority (EFSA) has implemented a molecular reference laboratory network for parasites such as*Echinococcus multilocularis*, enabling standardized testing and coordinated outbreak alerts across member states (Zancanaro & Papaleo, 2025). In the United States, the USDA’s National Animal Health Laboratory Network (NAHLN) integrates PCR and sequencing into national veterinary disease response programs, making molecular tools part of routine disease preparedness (Gamble et al., 2004).

In Latin America, regulatory frameworks are still developing but are gaining traction. In Peru, genomic surveillance of*pfhrp2/3*deletions in*Plasmodium falciparum*has directly influenced national malaria diagnostic policy. This example illustrates how molecular evidence can inform and guide public health decision-making (Cabrera-Sosa et al., 2024b).

Brazil occupies a strategic position in the region. With strong academic capacity and expanding laboratory infrastructure, Brazil is well placed to align molecular surveillance efforts with international standards. National studies have already demonstrated the role of molecular tools in zoonotic surveillance, particularly for *Leishmania infantum* (Cardoso et al., 2024), *Leishmania* spp. in wildlife (Ratzlaff et al*.*, 2022) and Toxoplasma gondii in food-producing animals (Lorenzi et al., 2016). This foundation provides a clear path for regulatory integration that could position Brazil as a regional leader in molecular policy frameworks.

### Challenges and limitations of molecular approaches

Although molecular tools have significantly advanced veterinary parasitology, their widespread application in routine diagnostics is still limited by practical challenges. High costs associated with equipment, reagents, sequencing platforms, and bioinformatics infrastructure remain a major barrier, particularly in low- and middle-income regions. In addition, the need for specialized training and technical expertise further restricts their implementation in many laboratories.

Another important limitation is the lack of standardization across methodologies. Variations in sample collection, DNA extraction, target selection, and data analysis make it difficult to compare results between studies and laboratories. This is especially relevant for metabarcoding and metagenomics, where differences in genetic markers, reference databases, and analytical pipelines can significantly influence outcomes. Moreover, next-generation sequencing approaches may be affected by contamination, low DNA input, and incomplete databases, while environmental DNA results require cautious interpretation due to the possibility of false positives and negatives.

Emerging technologies such as CRISPR-based diagnostics and lab-on-a-chip platforms show strong potential for rapid and portable detection, but they still require broader validation and standardization before routine use. Overall, the effective integration of molecular tools into One Health surveillance depends not only on technological advances but also on improved infrastructure, data sharing, trained personnel, and sustained funding to ensure reliable and reproducible results.

## Conclusions

Importantly, this review emphasizes that the impact of molecular tools is not determined solely by their technological sophistication, but by their accessibility and adaptability to different regional contexts. In Latin America, and particularly in Brazil, the successful integration of these approaches depends on overcoming structural barriers, strengthening laboratory capacity, and promoting technology transfer and training.

By explicitly addressing these challenges, this review contributes a region-focused perspective that complements existing global literature and highlights the need for more inclusive and context-adapted strategies in veterinary parasitology. This positioning reinforces the importance of bridging the gap between technological innovation and real-world implementation, especially in low- and middle-income regions.

However, despite these advances, critical challenges remain. Many studies still lack standardized protocols, reference genomes, and robust operational data. Infrastructure gaps, unequal access to sequencing technologies, and limited technical capacity in some regions create barriers to broad implementation. These limitations demand careful interpretation of current evidence and underscore the urgency of building more consistent, equitable, and standardized frameworks for molecular parasitology.

Looking ahead, the priority must be to standardize workflows, expand reference databases, conduct large-scale field validations, and integrate molecular findings with epidemiological and environmental data. Strengthening these pillars will allow veterinary services to move from reactive detection to proactive, precision-based surveillance and control. This shift will not only improve the early warning capacity for emerging and reemerging parasites but also reinforce global preparedness and response under the One Health framework.

## Data Availability

All data and information discussed in this review are derived from previously published studies and are available in the cited references throughout the manuscript. No new datasets were generated for this work.
